# The TIR Homologue Lies near Resistance Genes in *Staphylococcus aureus*, Coupling Modulation of Virulence and Antimicrobial Susceptibility

**DOI:** 10.1371/journal.ppat.1006092

**Published:** 2017-01-06

**Authors:** Sabine Patot, Paul RC Imbert, Jessica Baude, Patricia Martins Simões, Jean-Baptiste Campergue, Arthur Louche, Reindert Nijland, Michèle Bès, Anne Tristan, Frédéric Laurent, Adrien Fischer, Jacques Schrenzel, François Vandenesch, Suzana P Salcedo, Patrice François, Gérard Lina

**Affiliations:** 1 CIRI, Centre International de Recherche en Infectiologie, Inserm U1111, Université Lyon 1, Ecole Normale Supérieure de Lyon, CNRS UMR 5308, Lyon, France; 2 Laboratory of Molecular Microbiology and Structural Biochemistry, University of Lyon, CNRS UMR5086, Lyon, France; 3 Centre National de Référence des Staphylocoques, Hospices Civils de Lyon, Lyon, France; 4 Medical Microbiology, University Medical Center Utrecht, Utrecht, The Netherlands; 5 Laboratory of Phytopathology, Wageningen University, Wageningen, The Netherlands; 6 Genomic Research Laboratory, University of Geneva Hospitals, Geneva, Switzerland; Columbia University, UNITED STATES

## Abstract

Toll/interleukin-1 receptor (TIR) domains in Toll-like receptors are essential for initiating and propagating the eukaryotic innate immune signaling cascade. Here, we investigate TirS, a *Staphylococcus aureus* TIR mimic that is part of a novel bacterial invasion mechanism. Its ectopic expression in eukaryotic cells inhibited TLR signaling, downregulating the NF-kB pathway through inhibition of TLR2, TLR4, TLR5, and TLR9. Skin lesions induced by the *S*. *aureus* knockout *tirS* mutant increased in a mouse model compared with wild-type and restored strains even though the *tirS*-mutant and wild-type strains did not differ in bacterial load. TirS also was associated with lower neutrophil and macrophage activity, confirming a central role in virulence attenuation through local inflammatory responses. TirS invariably localizes within the staphylococcal chromosomal cassettes (SCC) containing the *fusC* gene for fusidic acid resistance but not always carrying the *mecA* gene. Of note, sub-inhibitory concentration of fusidic acid increased *tirS* expression. Epidemiological studies identified no link between this effector and clinical presentation but showed a selective advantage with a SCC*mec* element with SCC *fusC*/*tirS*. Thus, two key traits determining the success and spread of bacterial infections are linked.

## Author Summary

Pathogenic microbes have evolved elaborate strategies to manipulate host defenses to establish and spread in the host population. One such mechanism involves disruption of the immune signaling cascade orchestrated by the Toll-like receptors (TLRs), which sense microbial attack. TLR signaling elicits a proinflammatory response that controls immune cell recruitment to infected tissues. Here, we show that *Staphylococcus aureus*, an opportunistic human pathogen, expresses a host defense–like protein, TirS, that actively perturbs the initial TLR activation stage. Results with isolated human cells and mouse models show that TirS is a broad innate immune inhibitor of TLR-dependent signaling and modulates bacterial virulence, attenuating local inflammation. Moreover, the *tirS* gene lies near antimicrobial resistance genes for an antibiotic that enhances TirS production, shifting the balance to favor the pathogen and promote disease. Understanding mechanisms by which *S*. *aureus* modulates the immune response may lead to novel approaches for preventing and treating infection.

## Introduction

The innate immune system constitutes the first line of host defense against invading microbial pathogens in multicellular organisms. Key components of the innate immune response are pattern recognition receptors, which recognize a wide range of conserved bacterial structures, collectively called pathogen-associated molecular pattern and initiate an intracellular signaling immune cascade [1]. The Toll-like receptor/interleukin (IL)-1 receptor (TLR/IL-1R) superfamily, which comprises Toll-like receptors (TLRs) and interleukin-1 receptors (IL-1Rs), is required for many host innate immune responses and characterized by the presence of Toll/interleukin-1 receptor (TIR) domains cytoplasmically located on each TLR [2]. The TIR domain is critical for protein–protein interactions between TLRs with the corresponding TIR-containing adaptors. These interactions activate specific transcription factors such as nuclear factor-κB (NF-κB), which regulates the expression of various inflammatory mediators [3,4]. The TIR domain therefore plays a pivotal role in signaling from these receptors, and their importance in immune regulation has made them the subject of intense study.

The TLR signaling pathway is a key target of pathogen mechanisms of host immune system evasion [4]. Indeed, microbes can target various levels of the TLR signaling pathway, from modification of pathogen-associated molecular patterns to modifications in the immune signaling cascade. A potential host evasion mechanism involving TLRs came to light with the identification of bacterial TIR homologues. The majority of studies on bacterial TIR proteins have focused on their potential role as virulence factors that directly subvert host TLR signaling. For example, TIR-like protein A (TlpA) from *Salmonella enterica* serovar Enteriditis reduces NF-κB activation by a TLR4, IL-1R, and MyD88-dependent pathway and modulates IL-1β secretion during infection [5]. TcpC in the uropathogenic *Escherichia coli* CFT073 and Btp1/BtpA/TcpB in *Brucella* species suppress TLR2- and TLR4-mediated activation of NF-κB by targeting MyD88 [6,7]. A second TIR-containing protein in *Brucella* ssp. (BtpB) was reported to be a potent inhibitor of TLR signaling, probably via MyD88 as well [8].

The presence of a putative TIR–domain–containing protein in *Staphylococcus aureus* was suggested through a data search analysis [5] before being recently confirmed [9]. *S*. *aureus* is an important human pathogen that causes a wide variety of community and healthcare-associated infections [10]. This bacterium has a proven ability to adapt to the selective pressure of antibiotics. *S*. *aureus* was initially methicillin-sensitive (MSSA) but isolates resistant to this antibiotic were identified soon after its introduction (MRSA, or methicillin-resistant *S*. *aureus*) [11]. *S*. *aureus* becomes resistant to methicillin mainly by the acquisition of the methicillin-resistant gene *mecA*, which occurred first in hospital settings and now takes place in the community and in livestock [12,13]. The *mecA* gene is carried on a particular class of mobile genetic elements prevalent in staphylococci, the staphylococcal chromosomal cassette (SCC), designated as SCC*mec* [14]. Askarian et al. [9] characterized the TIR domain protein TirS in the SCC_476_ element of the methicillin-susceptible *S*. *aureus* strain MSSA476. SCC_476_ is integrated at the same site on the chromosome as SCC*mec* elements in MRSA [15]. TirS interferes with the TLR2-induced MAPK and NF-κB signaling pathway and enhances bacterial survival within the host [9].

In the present work, we report that TirS is spread among 12% of MRSA and MSSA strains. In an attempt to describe the genetic context of *tirS* (for staphylococcal TIR gene) in *S*. *aureus*, we fully sequenced the SCC element of representative bacterial strains. In all MRSA and MSSA lineages, the *tirS* gene was invariably located within this mobile genetic element and co-located with the *fu*s*C* and *mecA* (for the MRSA strains) antibiotic resistance genes. Interestingly, our results show that sub-inhibitory concentration of fusidic acid induced overexpression of *tirS*. We also confirm previous findings that *tirS* expression induces a negative regulation of the TLR signaling pathway. Our results with a mouse model of skin infection support that TirS modulates bacterial virulence through attenuation of host inflammatory responses during infection. This work is the first description of a TIR homolog protein carried by a mobile genetic element conferring resistance to antibiotics, suggesting a potential selective advantage. Indeed, these features may contribute to the ability of *S*. *aureus* to survive and establish a critical population size.

## Results

### *tirS* distribution and molecular epidemiology in *S*. *aureus* lineages

To assess the prevalence of *tirS* in various MRSA and MSSA lineages, a series of 226 well-characterized clinical isolates from more than 27 clonal complexes (CCs) or sequence types were subjected to *tirS*-specific PCR. Among the 226 strains examined, 28 (12.4%) yielded positive *tirS* amplification (Table 1). *tirS* was detected in MRSA and MSSA strains belonging to only 3 CCs: CC1, CC5, and CC8. In detail, the *tirS* gene was detected in 18/18 MRSA strains, CC5 Geraldine clone; 1/6 MRSA strains, CC5 pediatric clone; 1/2 MRSA strains, CC1; 6/9 MSSA strains, CC1; and 2/10 MSSA strains, CC8. Of further interest was our finding of a perfect association between *tirS* gene amplification and the MRSA Geraldine clone.

**Table 1:** Distribution of *tirS* gene among MSSA and MRSA clinical strains.

CC/ST ^a^^b^	SCC*mec* ^b^^c^	Clone name ^b^	No. of strains tested	No. of positive PCR tests for *tirS* /no. of strains tested (%)^d^
CC1	-	NA	9	6/9 (67)
	IV	USA400	2	0/2 (0)
	IV	WA MRSA-1/57	2	0/2 (0)
	V	Bengal Bay/WA MRSA-60	2	0/2 (0)
	V	Other	2	1/2 (50) *
CC5	-	NA	9	0/9 (0)
	**I**	**Geraldine**	**18**	**18/18 (100)** *
	IV	Pediatric	6	1/6 (16) *
	VI	New pediatric	2	0/2 (0)
	V	WA MRSA-11/80	2	0/2 (0)
	II	New York Japan	1	0/1 (0)
	II	EMRSA-3/Rhine-Hesse	3	0/3 (0)
CC8	-	NA	6	2/6 (40)
	I	North German/Iberian	3	0/3 (0)
	I	Ancestral	2	0/2 (0)
	III	Vienna/Hungarian/Brazilian	2	0/2 (0)
	IV	Lyon /EMRSA-2	6	0/6 (0)
	IV	EMRSA-14/WA MRSA-5	2	0/2 (0)
	IV	USA300	4	0/4 (0)
	IV	Other	2	0/2 (0)
	IV	MRSA-44	2	0/2 (0)
	IV	USA700	2	0/2 (0)
	V	MRSA-91	1	0/1 (0)
CC6	IV	WA MRSA 51	2	0/2 (0)
CC9	-	NA	1	0/1 (0)
CC10	-	NA	1	0/1 (0)
CC12	-	NA	3	0/3 (0)
CC15	-	NA	5	0/5 (0)
CC20	-	NA	2	0/2 (0)
CC22	-	NA	8	0/8 (0)
	IV	EMRSA-15/Barnim/Middle Eastern	5	0/5 (0)
CC30	-	NA	20	0/20 (0)
	IV	Southwest pacific	3	0/3 (0)
ST34	-	NA	4	0/4 (0)
ST36	II	EMRSA-16	2	0/2 (0)
CC45	-	NA	8	0/8 (0)
	IV	Berlin	2	0/2 (0)
	IV	Other	2	0/2 (0)
CC59	-	NA	3	0/3 (0)
	IV	USA1000	2	0/2 (0)
	V	Taiwan	2	0/2 (0)
	V	Other	1	0/1 (0)
CC80	IV	European	4	0/4 (0)
CC88	IV	WA MRSA-2	2	0/2 (0)
	V	Other	2	0/2 (0)
ST93	IV	Queensland Clone	2	0/2 (0)
CC97	-	NA	5	0/5 (0)
CC121	-	NA	5	0/5 (0)
CC130	XI	MRSA-XI	2	0/2 (0)
CC152	-	NA	5	0/5 (0)
CC182	-	NA	3	0/3 (0)
ST188	-	NA	2	0/2 (0)
ST398	-	NA	9	0/9 (0)
	IV	LA-MRSA	4	0/4 (0)
ST1755	-	*mecC*+	2	0/2 (0)
Other	-	NA	13	0/13 (0)

^*a*^ CC: clonal complex; ST: sequence type

^*b*^ CC/ST, SCC*mec*, and MRSA clone name were identified using the identibac *S*. *aureus* Genotyping Kit.

^*c*^—: absence of SCC*mec*

^*d*^ *tirS* gene detected by specific PCR as described in the Material and Methods

* SCC*mec* element sequencing

We next examined the molecular epidemiology of *tirS* in human staphylococcal infections. *S*. *aureus* strains were isolated from clinical specimens of individuals presenting skin and soft tissue infections, community-acquired pneumonia, bacteremia, infective endocarditis, or nasal colonization (asymptomatic bacterial carriage). No significant association was detected between specific disease and the presence of *tirS*. Among the 28 *tirS*-positive clinical samples, 10 were isolated from healthy patients (asymptomatic portage; 36%), 8 from patients with cutaneous infection (29%), 5 from patients with pneumonia (18%), 3 from patients with osteomyelitis (11%), 1 from a patient with bacteremia (4%), and 1 from a patient with infective endocarditis (4%).

### Genomic context of the *tirS* gene in MRSA and MSSA

**Fig 1:**
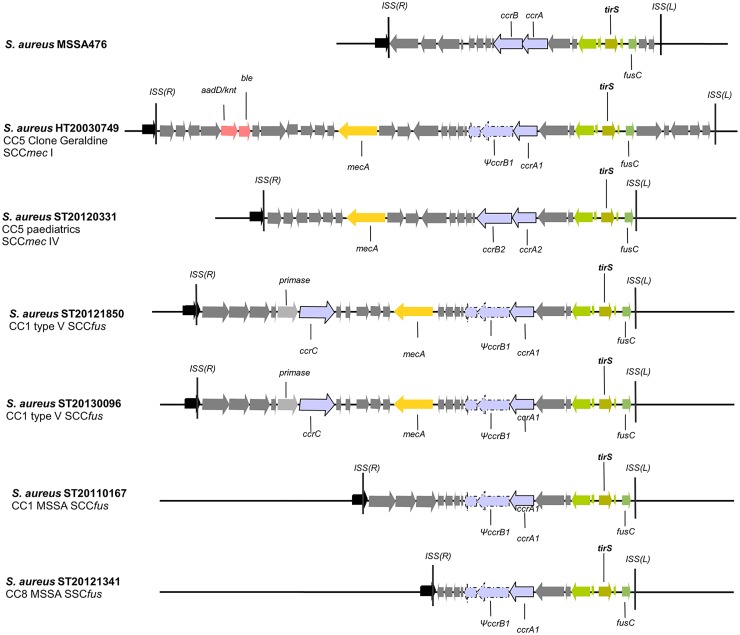
Characterization of the genomic context of the *tirS* region in *S*. *aureus* MRSA and MSSA strains. Localization of the *tirS* gene and surrounding context in 4 *tirS*-positive MRSA strains and 2 *tirS*-positive MSSA strains. For all 6 strains, the *tirS* gene was found within the SCC element. Schematic diagrams represent the genomic comparison of the 6 SCC regions of both MRSA and MSSA strains used in our study and the SCC_476_ element of strain MSSA476 for which the *tirS* gene was first described [9]. Predicted ORFs are marked in the direction of transcription as arrows. The highly conserved *tirS* region, consisting of the *tirS* gene and 4 surrounding genes among which we find the *fusC* gene (fusidic acid resistance), is represented by green arrows. Light rose arrows represent genes coding for antibiotic resistance, orange arrows represent the *mecA* gene conferring resistance to beta-lactams and blue-gray arrows represent the site specific recombinases (*ccr*AB or *ccr*C) of SCC elements. The SCC elements ISS(L) and ISS(L)) are also represented as black vertical bars. Abbreviations: *ccr*AB = cassette chromosome recombinase A and B; *fusC* = fusidic acid resistance gene; *mecA* = methicillin resistance gene; *ccrC*: cassette chromosome recombinase C; *ble* = bleomycin resistance gene; *aaD/knt* = kanamycin nucleotidyltransferase (resistance gene).

Because the *tirS* gene was previously described on a staphylococcal chromosomal cassette SCC_476_ [9], we performed whole-genome shotgun sequencing of six representative MRSA and MSSA strains positive for the presence of the *tirS* gene. These included four MRSA lineages: the prototype Geraldine clone strain HT20030749 (CC5; SCC*mec* I), strain ST20120331 (CC5, SCC*mec* IV), and strains ST20121850 and ST20130096 (CC1; SCC*mec* V *fusC+*) (ENA database project study accession number PRJEB12840; sample accessions ERS1070204 to ERS1070207). Two MSSA strains *tirS*-positive were also added to the study: strain ST20110167 (CC1) and strain ST20121341 (CC8) (sample accessions ERS1434451 and ERS1434452). As reference to the comparison with our sequenced strains, we used the genome of strain MSSA476, in which *tirS* was recently described [9]. Whole-genome alignment and search for the site-specific insertion sequences (ISS) typical of SCC-like cassette insertions showed that *tirS* is invariably present within the SCC element of all six MRSA and MSSA genomes analyzed (Fig 1). Moreover, this gene is found in a highly conserved region within the J1 region (between the *ccr* complex and the 5' ISS(L)), consisting of five open reading frames (ORFs) that include the *tirS* gene but also the *fusC* gene, responsible for resistance to fusidic acid. This region is present as well in MRSA as in the MSSA strains analyzed (Fig 1).

Furthermore, BLASTn searches using the *tirS* and its surrounding four ORFs (“*tirS* region”), as annotated in the strain MSS476 genome (GenBank: BX571857.1), against the GenBank nucleotide collection (nr/nt) revealed the presence of the *tirS* region in 14 MRSAs, 2 other MSSAs, and a methicillin-sensitive *Staphylococcus hominis* strain. Unexpectedly, in all 17 staphylococci, the *tirS* region was conserved and located in SCC elements in the vicinity of the site-specific recombinase of type *ccr*AB. For 15 of the *S*. *aureus* strains in which the *tirS* region was detected, a whole-genome shotgun sequence was available, and we observed that they belonged to four distinct clonal complexes: CC1 (2 MRSA and 1 MSSA), CC5 (7 MRSA), CC8 (1 MSSA), and CC22 (4 MRSA).

### TirS production

**Fig 2:**
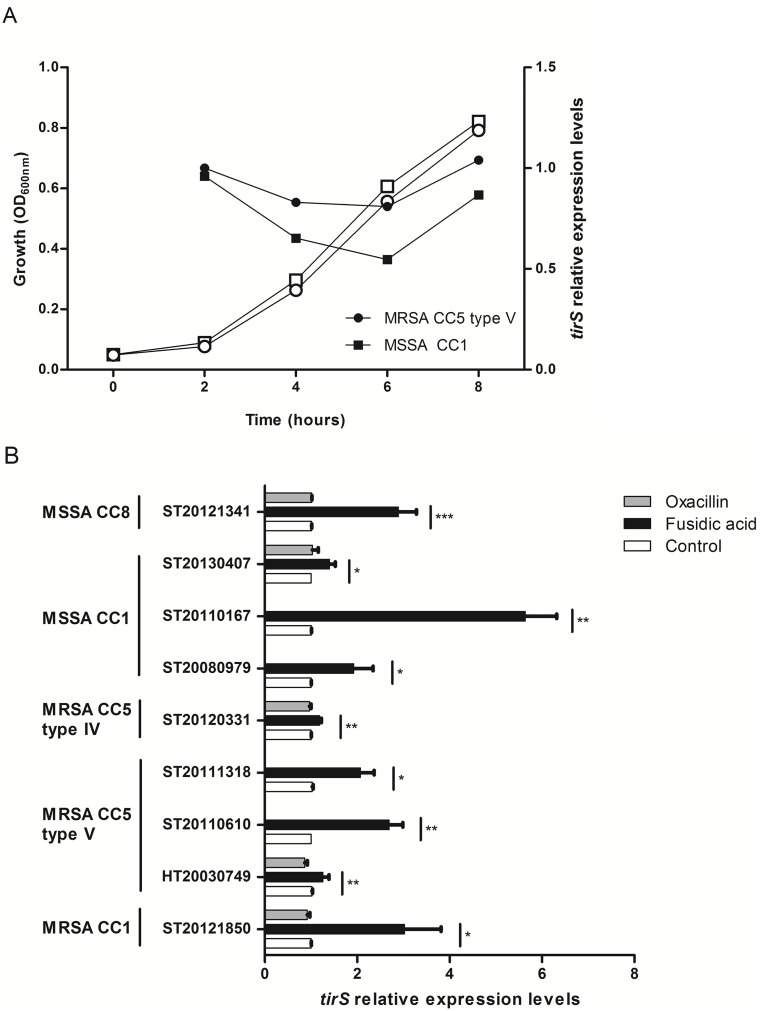
Impact of bacterial growth and antibiotic on *tirS* expression. (A) The expression of *tirS* was quantified by RT-qPCR and normalized to the level of *hu* expression from total RNA extracts prepared from bacterial cultures at 2, 4, 6, and 8 h. MHB medium condition at 2 h was used as a reference. The figure shows an average of 2 independent experiments. White symbols represent bacterial growth, black symbols represent *tirS* expression. (B) Expression of *tirS* on 2-h bacterial cultures and 30 min of antibiotic treatment. Bacterial strains without stress were used as reference (relative quantity = 1) to estimate the relative quantity of *tirS* mRNA. White bars correspond to negative control (no antibiotic), grey bars to bacteria exposed to oxacillin, and black bars to bacteria exposed to fusidic acid. Data represent mean ± SEM of three to six independent assays. * p < 0.05; ** p < 0.01; *** p < 0.001.

To investigate TirS production by *S*. *aureus*, *tirS* expression was examined by quantitative real-time reverse transcription PCR (RT-qPCR) during bacterial growth in one MRSA (clone Geraldine, CC5) and one MSSA (CC1) *tirS*-positive strains. The analysis of the *tirS* expression kinetics showed stable expression of *tirS* during growth with a marginal increase at the end of the exponential phase (Fig 2A). The difference in *tir*S expression and the standardization gene *hu* was generally around seven cycles.

Because *tirS* is located in the SCC element, we investigated the effect on *tirS* expression of adding a sub-minimum inhibitory concentration (sub-MIC) of oxacillin (1/2 MIC) and fusidic acid (1/4 MIC) during the exponential phase of *S*. *aureus* growth in 9 strains. Addition of fusidic acid appeared to significantly modulate *tirS* expression both in MSSA and MRSA strains (Fig 2B). *tirS* was upregulated up to six-fold with fusidic acid compared to its expression without antibiotic stress and level of induction by fusidic acid was strain dependent. In contrast, no difference in levels of expression of *tirS* was observed with exposure to sub-MIC of oxacillin.

### TirS interferes with TLR signaling

**Fig 3:**
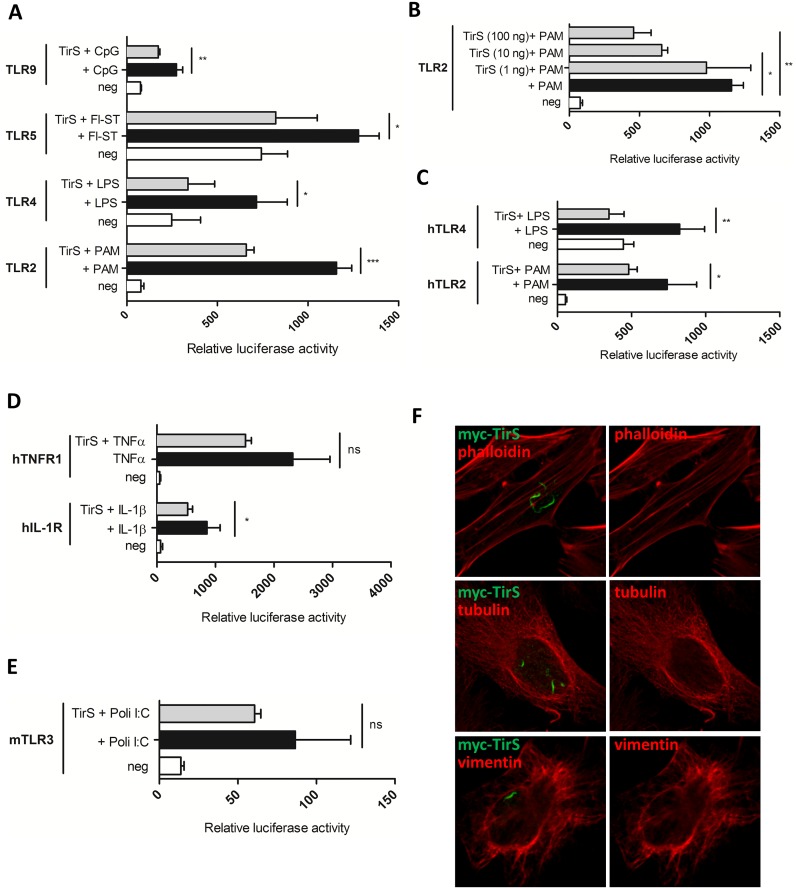
TirS interferes with TLR and IL-1R signaling. (A) HEK293T cells were transiently transfected for 24 h with the luciferase reporter vector and murine TLR2, TLR4, TLR5, or TLR9 in the presence or absence of TirS (50 ng). Cells were then stimulated with the appropriate ligand (PAM, LPS, Fl-ST, and CpG) for 6 h before measurement of luciferase activity. White bars correspond to negative control, black bars to cells stimulated with the appropriate ligand, and gray bars to cells transfected with TirS and stimulated with the ligand. Data represent the means ± SEM of the relative luciferase activity and were obtained from duplicates of 3 independent experiments. (B) Luciferase activity of murine TLR2 following transfection with increasing amounts of vector encoding *tirS* (1, 50, 100 ng) in order to obtain different levels of expression and for (C) human TLR2 and TLR4. (D) Effect of TirS on TNFR and IL-1R activation following TNF-α or IL-1β stimulation, respectively and (E) TLR3 following stimulation with poli:IC. (F) HeLa cells were transfected with myc-TirS for 10 h and labeled for myc (green) and different components of the cytoskeleton: actin (top panels, phalloidin), microtubules (medium panels, tubulin), or intermediate filaments (bottom panels, vimentin). ns: not significant, * p < 0.05, ** p < 0.01, and *** p < 0.001.

TirS belongs to the family of bacterial TLRs [9]. We therefore investigated the ability of TirS to specifically interfere with TLR signaling using an *in vitro* NF-κB-dependent luciferase reporter system. Although a variety of TLR receptors have been described, in humans the most relevant for recognition of bacterial molecules are TLR2, TLR4, TLR5, and TLR9. Ectopic expression of *tirS* in HEK293T cells transfected with the luciferase reporter vector and various TLRs resulted in reduced TLR2 activation and to a lesser extent, reduced activation of TLR9, TLR4, and TLR5 (Fig 3A). As a control, we confirmed that this inhibitory effect of TirS on murine TLR2 was dose-dependent by carrying out the transfections with increasing amounts of the expression vector encoding TirS (Fig 3B). Inhibition of human TLR2 and TLR4 pathways following addition of the appropriate ligands was also observed (Fig 3C). These results suggest that TirS may, at least partly, target a common molecule between these pathways such as the adaptor molecule MyD88. Consistently, reduction of IL-1R following IL-1β stimulation was also observed in the presence of TirS (Fig 3D). In contrast, although much lower levels of activation can be obtained for TLR3, which is independent of MyD88, we did not observe any significant effect of TirS on this pathway (Fig 3E). This was also the case for endogenous TNF receptor (TNFR) (Fig 3D). Overall, these results are in agreement with data from Askarian et al. [9] that previously described TirS inhibition of TLR2, MyD88 and TIRAP dependent pathways *in vitro*.

In addition to its ability to interfere with TLR signaling, the bacterial TIR effector protein from *Brucella* BtpA targets and modulates microtubules [16] through a WxxxE motif [17] as well as the recently identified TIR protein from *Bacillus anthracis* referred to as BaTcp [18]. Since the WxxxE is also present in TirS, we investigated the intracellular localization of ectopically expressed TirS by confocal microscopy on HeLa cells transfected with myc-TirS and indirect immuno-fluorescence staining with anti-myc antibodies. We found that myc-tagged TirS accumulated in filament-like structures of irregular shapes within the host cytosol (Fig 3F). Similar results were observed for GFP-TirS, suggesting that this localization was not dependent on the tag (S1 Fig). In addition, TirS showed no co-localization with cytoskeleton components as observed after labeling with either phalloidin for actin, tubulin for microtubules, or vimentin for intermediate filaments (Fig 3F), suggesting a different targeting than previously described for BtpA and BaTdp.

### TirS modulates *S*. *aureus* virulence

**Fig 4:**
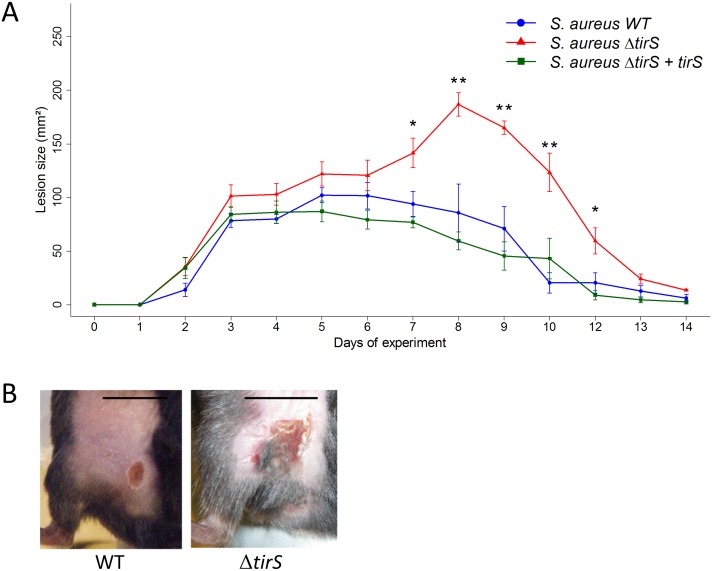
*S*. *aureus* deleted for *tirS* induced larger skin lesions in WT C57Bl/6 mice. (A) Data are presented as mean total lesion size (mm^2^) ± SEM and are representative of 3 independent experiments with at least 4 mice/group. * p < 0.05; ** p < 0.01 (B) Photographs of representative lesions at day 7 after staphylococcal infection. Black bars indicate 10 mm. Abbreviations: WT = wild-type; Δ*tirS* = deleted for the *tirS* gene; Δ*tirS* + *tirS* = Δ*tirS* strain chromosomally restored for *tirS* gene.

To further investigate the role of TirS during infection, we carried out *in vivo* studies in mice. Because *S*. *aureus* is the leading cause of skin infection, we developed a subcutaneous model of infection in mice with the inoculation of *S*. *aureus* clone Geraldine-wild type (WT) strain, a deleted for the *tirS* gene (Δ*tirS*) strain, or a Δ*tirS* strain restored for *tirS* gene in a chromosomic position (Δ*tirS* + *tirS*). First, we confirmed that bacterial growth was not affected by genetic manipulation of TirS by assessing growth in two different media (brain–heart infusion (BHI), tryptic soy broth (TSB)) (S2 Fig). Then, wild-type (WT) C57Bl/6 mice were inoculated with the three strains. Cutaneous infection resulted in the development of visible lesions by day 1 that healed by day 14 regardless of the strain (Fig 4A and 4B). As control, injection of sterile phosphate buffered saline (PBS) did not induce any skin lesions in mice. Infection with Δ*tirS* strain resulted in larger lesions (~2.5-fold) compared with the WT strain (p < 0.01). These differences appeared around day 6 and persisted for about 4 days before being resolved at the same time for both strains. Similar lesion sizes were observed in mice infected with the *S*.*aureus* restored strain compared with the WT strain (p = 0.9), confirming the role of TirS on the observed results.

**Fig 5:**
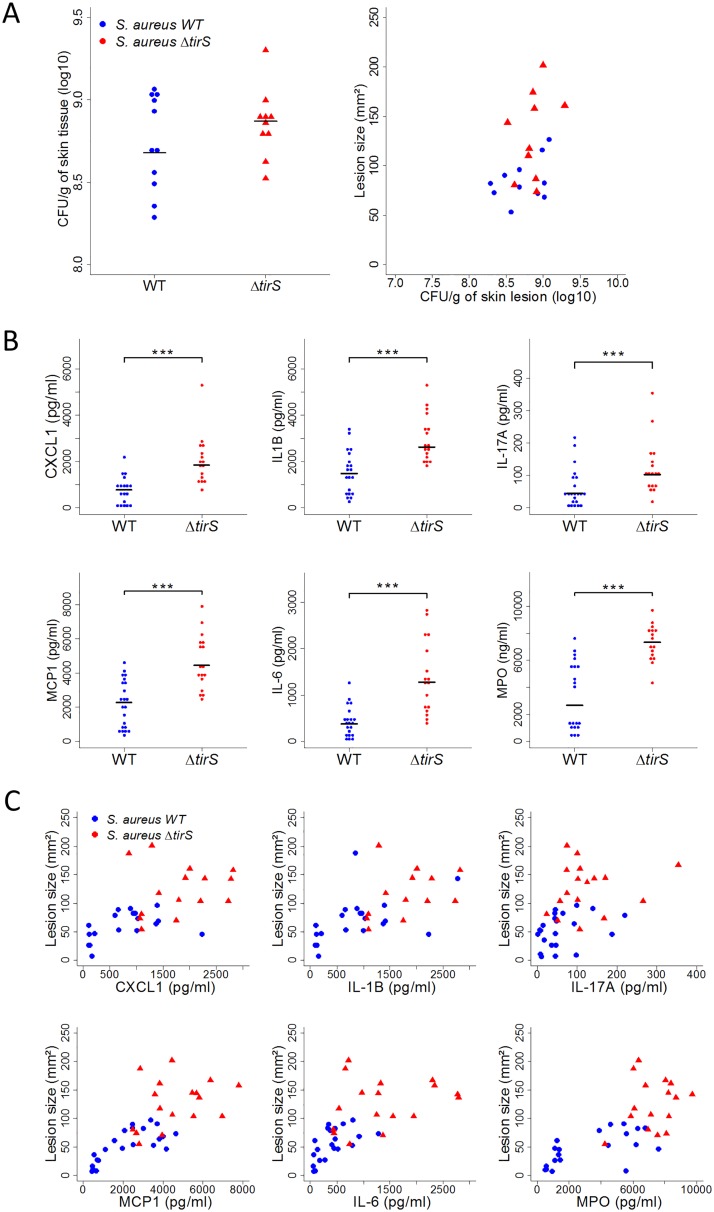
TirS induces reduced inflammation despite similar bacterial counts in skin lesions. (A) Bacterial burden in skin lesions of mice infected with *S*. *aureus* WT (n = 11) or *S*. *aureus* Δ*tirS* (n = 10) and correlation between bacterial burden and lesion size. Data were obtained 5 and 7 days after inoculation from 2 independent experiments. (B) Levels of IL-1β, IL-6, IL-17, CXCL1, MCP1 (pg/ml), and myeloperoxidase (MPO) activity (ng/ml) from lesional skin at 7, 8, and 9 days after inoculation. Data were obtained from 16 to 22 mice infected with *S*. *aureus* WT or *S*. *aureus* Δ*tirS* from 4 independent experiments (all data shown). (C) Correlation between levels of IL-1β, IL-6, IL-17, CXCL1, MCP1, MPO, and lesion size from lesional skin at 7, 8, and 9 days after inoculation. Data were obtained from 16 to 22 mice infected with *S*. *aureus* WT or *S*. *aureus* Δ*tirS* from 4 independent experiments (all data shown). Each symbol represents an animal and the median values are marked by horizontal bold lines. Abbreviations: WT = wild-type; Δ*tirS* = deleted for the *tirS* gene. *** p < 0.001.

To better understand the factors that might be important in determining the size of skin lesions in the mouse model, animals were sacrificed between 5 and 9 days after infection induced by MRSA clone Geraldine WT and Δ*tirS* strains to the sample lesion. First, we enumerated the bacterial load in the skin lesion. No significant differences in colony-forming unit (CFU) counts between the strains were observed (Fig 5A). Moreover, lesion size was not correlated with bacterial burden in either the WT or Δ*-tirS* strain (*S*. *aureus* WT: r^2^ = 0.39; p = 0.2, *S*. *aureus* Δ*tirS*: r^2^ = 0.35; p = 0.2) (Fig 5B). This finding raised the possibility that the inflammatory response was more important in determining lesion severity, as assessed by lesion size, than bacterial burden in the lesions.

To follow up on these observations, we sought to measure neutrophil and macrophage activity with the quantification of levels of myeloperoxidase (MPO) and a panel of inflammatory cytokines in the skin lesions of mice infected with MRSA clone Geraldine WT and Δ*tirS* strains. Interestingly, the levels of MPO and IL-1β, IL-6, IL-17, CXCL1, and MCP1 were significantly lower in the murine skin lesions developed after the WT strain inoculation compared with the Δ*tirS* strain (Fig 5B). Correlation between skin lesion size and the levels of MPO, IL-1β, IL-6, IL-17, CXCL1, and MCP1 was statistically confirmed for each of these inflammatory markers (MPO: r^2^ = 0.71, p < 0.001; CXL1: r^2^ = 0.55, p < 0.001; IL-1β: r^2^ = 0.61, p < 0.001; IL-17A: r^2^ = 0.45, p < 0.01; MCP1: r^2^ = 0.74, p < 0.001; IL-6: r^2^ = 0.64, p < 0.001) (Fig 5C). Therefore, the smaller lesions observed after experimental inoculation with the bacterial WT strain were associated with lower levels of chemokines and less inflammation but not with decreased bacterial burden. These data support the notion that the local inflammatory response is a key determinant of lesion size. These results are consistent with a role for TirS in the control of the inflammatory response during *S*. *aureus* infection *in vivo*.

**Fig 6:**
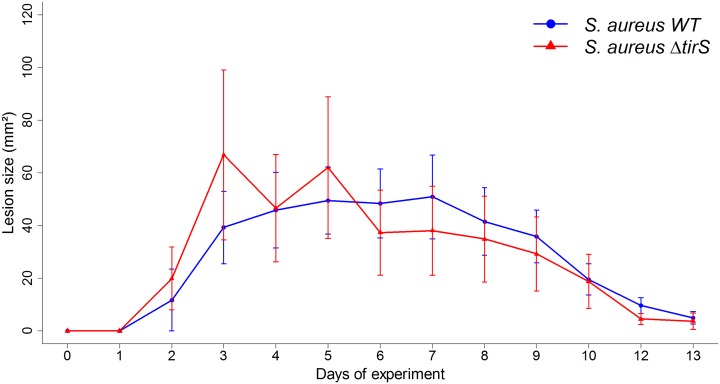
*S*. *aureus* deleted for *tirS* induced similar skin lesion size in MyD88-deficient mice. Data are presented as mean total lesion size (mm^2^) ± SEM and are representative of 2 independent experiments with at least 4 mice/group. Abbreviations: WT = wild-type; Δ*tirS* = deleted for the *tirS* gene.

Finally, to evaluate the contribution of the TIR-containing adaptor protein MyD88, a key component of the TLR signaling pathway implicated in proinflammatory mechanisms [19], MyD88-deficient mice from the C57BL/6 genetic background were subcutaneously inoculated with MRSA clone Geraldine WT and Δ*tirS* strains. An identical infection protocol and bacterial concentration were used as in WT mice. As control, injection of sterile PBS did not induce any skin lesions in mice. Interestingly, there was no significant difference in lesion size between MyD88-deficient mice infected with the WT or Δ*tirS S*. *aureus* strain (Fig 6), suggesting that MyD88 might play a role in virulence of the MRSA clone Geraldine used. These results confirm *in vitro* previously reported data showing that TirS inhibits signaling in a MyD88-dependent manner [9].

## Discussion

Bacterial strategies for innate immune evasion involve manipulation of the TLR signaling by TIR homologues such as TirS for *S*. *aureus* [20]. In this work we report the localization of the *tirS* in different SCC elements and its role in the control of the inflammatory response during *S*. *aureus* infection. Using a mouse model of *S*. *aureus* skin infection, we evaluated the role of TirS on *S*. *aureus* virulence. We show that *S*. *aureus* Geraldine deleted for the *tirS* gene exhibited superior virulence compared to the WT strain, as attested by the size of the skin lesion. Of note, bacterial counts in the skin lesions did not differ between the mutant and the WT strains and did not correlate with clinical severity (*i*.*e*., lesion size). This finding suggests that bacterial burden may not be the primary driver of lesion severity and argues that lesion severity may be due, at least in part, to the associated inflammatory response. In support of this hypothesis, we found a correlation between lesion size and levels of the proinflammatory cytokines, as well as neutrophil activity (as assessed by MPO levels). These findings are consistent with previous studies, which underscores that the severity of skin infection is often driven by the inflammatory response to the invading pathogen as much or more than by the direct effects of the pathogen itself [21–23]. This inference suggests that the attenuation of skin inflammation observed with *S*. *aureus* WT strain, compared to its *tirS* mutant, was driven by modulation of the inflammation resulting from TirS action. Such a conclusion is concordant with the fact that the ectopic expression of TirS in eukaryotic cells appeared to temper stimuli-induced TLR2-, TLR4-, TLR5-, and TLR9-mediated NF-kB activation. Accordingly, a previous and independent work has reported a negative interference of TirS with the TLR2 signaling pathway [9]. These results can be directly linked to our *in vivo* observations in mice, explaining the modulation of virulence during *S*. *aureus* infection by *tirS* [4,20]. Here the bacterial TIR effector has been shown to induce attenuation of virulence during infection because of the downregulation of the innate immune pathway.

In addition to control of inflammatory responses, some TIR domain-containing proteins such as BtpA or BaTcp have been shown to target and modulate microtubules [18,24] through a WxxxE motif that is also present in TirS and involved in cross-talk between the TLR and GTPase signaling pathways [16]. We found that ectopically expressed TirS accumulated in filament-like structures of irregular shapes within the host cytosol but that do not co-localize with typical cytoskeleton components (microtubules, actin or intermediate filaments). This observation is different from those previously described for other bacterial TIR proteins from *Brucella* [8,16] and BaTdp from *B*. *anthracis* [18]. The role of TirS accumulation in filament-like structures in the modulation of *S*. *aureus* pathogenicity remains to be explored.

The host interacting partner of TirS has yet to be identified. MyD88 is a general adaptor protein that plays an important role in Toll/IL-1 receptor family signaling. *In vitro* results of Askarian et al. [9] showed that TirS interferes with TLR2 and both MyD88 and TIRAP pathways *in vitro*. Here we report that *in vitro*, TirS is a potent inhibitor of not only TLR2 but also TLR4, TLR5, and TLR9. All of these proteins are dependent on MyD88, a general adaptor protein that plays an important role in the Toll/IL-1 receptor family signaling, which argues for an interaction of TirS with MyD88. Consistently, we found that *S*. *aureus* carrying *tirS* induced no increased virulence in a MyD88 knockout mice model. Taken together, these results suggest that TirS modulates an inflammatory response at the site of the infection through the MyD88 adaptor protein. Preliminary work from our lab has not been able to purify TirS, which is highly insoluble and failed to detect an interaction by co-immunoprecipitation assays. Further work is now required to understand the molecular mechanism by which TirS interacts and/or competes with MyD88.

As yet, there is no clear consensus on the mechanism by which TIR proteins enter host cells and localize to the host cell cytoplasm. From deduced amino acid sequences, no recognizable signal sequence for secretion was detected in TIR proteins, including TirS. In the case of TlpA from *Salmonella enterica*, for which no direct evidence of secretion has been reported, the suggested mechanism is a role for type III or type IV secretion systems (T3SS or T4SS) that can directly inject TIR effectors into the host cells [5]. TcpC of *E*. *coli* has been reported to be secreted into the media of cultured bacterial cells and subsequently taken up into host macrophages to interfere with TLR-mediated tumor necrosis factor induction [17]. In the case of *Brucella*, BtpA and BtpB are translocated into host cells in a manner partially dependent on the T4SS [8]. As observed with other TIR protein genes, the *tirS* gene is not co-located with genes encoding for a secretion system in *S*. *aureus*. However, TirS has been reported to be secreted into the media through an unknown mechanism [9]. Further studies are needed to clarify this issue, perhaps by performing real-time imaging and co-localization studies.

A comparison of different SCC elements in various *S*. *aureus* genetic backgrounds highlights the invariable presence of *tirS* within the SCC *fusC*/*tirS* mobile genetic element, sometimes included in the SCC*mec* elements in the J1 region. As proposed previously [9, 25], the finding that *tirS* region was invariably present within an SCC element suggests that similar to *mecA* and *fusC* transmission, SCC-mediated horizontal transfer is the major mechanism of *tirS* dissemination. Moreover, horizontal transfer of the *tirS* region is also suggested with the nearby presence of site-specific recombinases of the *ccr*AB type in all SCC elements carrying the *tirS* gene [25]. Of further interest, all published sequences of SCC *fusC*/*tirS* have identified four conserved additional genes. Future work should attempt to assign functional roles of these putative proteins and evaluate their involvement in the regulation or modulation of *tirS* and *fusC* transcription.

The co-location of *mecA* and *fusC* genes in this genetic element suggested that, in addition to antibiotic resistance genes, the presence of *tirS* may also confer a selective advantage for *S*. *aureus*. However, our observation indicated that a sub-inhibitory concentration of fusidic acid but not oxacillin induced overexpression of *tirS* in all strains tested. These results were unexpected because we observed the opposite outcome for Panton Valentine leukocidine and alpha-toxin expression with the same antibiotics: overexpression by oxacillin and inhibition by fusidic acid [26]. The acquisition by *S*. *aureus* of a gene encoding for factors that modulate virulence in the SCC*mec* element is not exceptional. The *pls* gene encoding for a large surface protein with an LPXTG peptidoglycan-anchoring sequence is a part of the type I SCC*mec* element [27]. This protein reduces *S*. *aureus* adherence and invasiveness [28]. Pls was also described in SCC*mec* III [29] but never in MSSA. Conversely, genes encoding for phenol soluble modulins alpha 1 to 3 (PSM-α1–3; small peptides with an amphipathic α-helical structure and strong surfactant-like properties that induce the production of proinflammatory cytokines and recruit, activate and lyse neutrophils) were first described in MSSA [30] before being found to be part of SCC*mec*II, SCC*mec*III, and SCC*mec*VIII (PSM-α*-mec*) [31,32]. To our knowledge, is it not yet known whether antibiotics whose resistance is encoded by these SCC elements modulate the expression of Pls and/or PSM-*mec*.

A number of bacteria expressing TIR-containing proteins have been described, but as far as we know, only one study references their prevalence throughout a bacterial species [24]. In this work, we report the presence of *tirS* gene in 12.4% of a clinical MSSA and MRSA *S*. *aureus* strain collection. The TirS effector was not shown to be associated with a specific clinical human presentation by molecular epidemiology studies. For the Geraldine clone that always holds the *tirS* gene, previous observation did not identify a particular association with disease severity [33,34]. By contrast, the first nosocomial outbreak due to the Geraldine clone was recently reported, emphasizing its efficiency in being transmitted and easily spread within health care settings [35]. Indeed, this clone has been reported to be both a community-acquired and hospital-acquired MRSA. Thus, the clone Geraldine SCC*mec* element may provide a selective advantage in both settings: in the hospital because of a higher antimicrobial resistance compared to drug-susceptible WT *S*. *aureus* strains, and in the community because of its enhanced inhibition of the innate immune response. Similarly, different groups recently described the emergence in the community and in the hospital of ST1, ST45, and ST149 MRSA *fusC* positive strains in England and of *fusC* positive ST5 MRSA in New Zealand that also are *tirS* positive [36,37]. These epidemiological observations highlight the selective advantage of *S*. *aureus* in carrying the SCC*mec* element with SCC *fusC*/*tirS*.

In summary, we identify for the first time a bacterial TIR homolog protein genetically linked to an antimicrobial agent resistance determinant in the genetic mobile element SCC, thus providing a molecular connection between two key traits determining the successful outcome and spread of bacterial infections. Moreover, TIR homolog protein production was modulated by one antibiotic, fusidic acid, for which the resistance is encoded in a conserved region, which includes the *tirS* gene, and located within these SCC*mec* and non-*mec* SCC elements. This result expands knowledge about bacterial TIR homologs that constitute an ingenious strategy of pathogenic bacteria to evade the host immune system. The current state of knowledge strongly suggests that TIR effectors should be considered as potential key effectors of host defense, which emphasizes that further research is required to elucidate the precise mechanism of action of these interesting molecules. From the clinical point of view, the identification of the critical role of TirS signaling for modulating the immune response to a site of infection raises the possibility that this pathway could be locally targeted to engage the host’s own immune responses in the treatment of a microbial infection.

## Materials and Methods

### Ethical statements

Isolates were obtained as part of routine diagnostic testing and were analyzed anonymously. All data were collected in accordance with the European Parliament and Council decision for the epidemiological surveillance and control of communicable disease in the European community [38,39]. Ethical approval and informed consent were not required.

All mouse protocols were carried out in strict accordance with the Directive 2010/63/EU revising Directive 86/609/EEC on the protection of animals used for scientific purposes. This directive was translated in the French regulation as the Décret N°2013–118 of February 2013 under the jurisdiction of the Ministry of Education, Research and Technology. The initial research project had been approved by the local Animal Ethic Evaluation Committee CECCAPP (Comité d’Evaluation Commun au Centre Léon Bérard, à l’Animalerie de transit de l’ENS, au PBES et au laboratoire P4) with the references ENS_2014_025 and ENS_2014_052 and subsequently authorized by the French Ministry of Education, Research and Technology.

### Bacterial strain characterization

A subset of 226 strains from the collection of the Centre National de Référence des Staphylocoques (Lyon, France), composed of 103 strains of the main community and hospital-acquired MRSA clones, and 123 strains of MSSA were used in this study. They were sent to the laboratory for detection of toxin production in the context of nasal colonization, skin and soft tissue infection, pneumonia, bacteremia, or endocarditis. The *S*. *aureus* HT20030749 strain belonging to the clone Geraldine was isolated from blood culture of patient with bone–joint infection.

All strains were genotyped as previously described. Briefly, bacterial DNA was extracted according to the manufacturer's recommended protocol using commercial extraction kits (Qiagen). The diagnostic DNA microarrays, identibac *S*. *aureus* Genotyping (Alere) used for this study, as well as related procedures and protocols, have been previously described in detail [40]. This microarray covers 332 different target sequences corresponding to approximately 185 distinct genes and their allelic variants. The assigning of isolates to CCs was determined by the comparison of hybridization profiles with those previously characterized by using multilocus sequence typing reference strains [40].

#### Illumina Sequencing

Genomic DNA were extracted from each isolate using a QIAcube extraction kit (Qiagen). The Nextera XT DNA preparation kit (Illumina) was used to generate sequencing libraries from 1 ng of DNA. Whole-genome sequencing was finally done with an Illumina HiSeq (Illumina, San Diego, CA, USA) to generate 150-bp paired-end reads.

#### *De novo* assembly

For each isolate, the raw paired-end reads were assembled using a modified version of the A5-miseq open-source pipeline [41]. This pipeline implements a complete sequencing data processing workflow from raw read cleaning to *de novo* assembly. The first task of the read cleaning involves removing the regions of the raw reads that are contaminated by the adapters during the Nextera XT protocol using the Trimmomatic program [42]. Then, the reads are filtered and trimmed according to quality and length criteria using Trimmomatic and the preprocess function of String Graph assembler (SGA) [43]. Finally, the correct function of SGA is used to correct errors in the reads by a k-mer frequency-based method. After being quality filtered and error corrected, the reads were assembled by the IDBA-UD500 program, which implements an iterative De Bruijn graph built with several values of k-mer lengths, from low to high values, instead of a single value as for most *de novo* assemblers [44]. The reads are then mapped against the assembly using BWA-MEM [45] to polish the contigs at every position where basecalls differ between the mapping and the assembly. The scaffolding implemented at the end of the original A5-miseq pipeline was not performed because it provides no gain in the subsequent analysis of marker detection.

#### Characterization of SCC*mec* V and *tirS* regions

First, the three MRSA genomes were aligned against the strain MSSA476 genome using progressiveMauve with default parameters [46]. Then, identification of SCC was done by searching for the ISS of these elements [47,48], both at the end of the ORF X / *rlmH* gene and further downstream. The newly detected SCC*mec* elements were annotated using the RAST Server (http://rast.nmpdr.org/), and blastn searches were performed (http://blast.ncbi.nlm.nih.gov/) against the nucleotide collection (nr/nt) using the megablastn algorithm and the organism filter for *S*. *aureus* (taxid:1280).

### *tirS* amplification

Oligonucleotide primers *tirS*-For-CTTCAAAAAGAGCAGTCTAGG and *tirS*-Rev-CTTCAACACTCACTTTATGCC were designed according to *tirS* sequence. After amplification for 30 cycles (30 s of denaturation at 94°C, 30 s of annealing at 53°C, and 30 s of extension at 72°C), the PCR products were resolved by electrophoresis through 1.5% agarose gels (Sigma, Saint Quentin Fallavier, France). This step was followed by SYBR Safe DNA (Life Technologies) staining and analysis. To assess the specificity of *tirS* amplification, PCR products were subjected to DNA sequencing (Biofidal, Lyon, France). *S*. *aureus* HT20030749 and RN4220 strains were used as positive and negative amplification controls, respectively.

### Transcription of *tirS*

*tirS* expression was analyzed using RT-qPCR. MRSA ST20121850 (CC1), MSSA ST20130407, ST20110167, ST20080979 (CC1), MSSA ST20121341 (CC8), MRSA ST20120331 (CC5, type IV), MRSA ST20111318, ST20110610, HT20030749 (CC5, type V) were grown in fresh MHB at 37°C, after a 1:100 dilution of overnight cultures. For kinetics analysis, total RNA of two representative strains (MRSA HT20030749, CC5 and MSSA ST20130407, CC1) was purified at 2, 4, 6, and 8 h of growth as previously described [49]. Total RNA was extracted with the RNeasy Plus (Qiagen) kit including a gDNA eliminator column and an additional DNAse treatment (Qiagen). RNA quality and quantity were determined by Bioanalyzer (Agilent) using the RNA Nano chips and quantified using the ND-8000 (NanoDrop Technologies). Absence of DNA contamination was checked by using *tirS*-specific primers and probe at optimal concentrations (assessed as previously described [50] without the reverse-transcription step). The final concentrations were 0.2 μM for primers and probe (*tirS*-F-CTATTTGGCATAAAGTGAGTGTTGAAG, *tirS*-R-AAATCACTTGTATTCAATGCATACTTATCT, and *tirS*-P-CGTGCATACAACCCATAT labeled with NED at the 5’ and 3’-minor groove binder), and reactions were performed in a one-step RT-PCR enzymatic mixture (Agilent Technologies, Brilliant II QRT-PCR Master Mix Kit) in a final volume of 20 μl in the CFX96 system (Bio-Rad) and following manufacturer’s instructions. Differences in Ct values between tested transcripts and *hu* signals were used for normalization purposes and based on the MHB medium condition at 2 h as a reference. The fold change was expressed as the inverse exponential of the difference between MHB Ct (reference) and the stress condition Ct. This assay was also used on 2-h cultures to assess gene expression values of *tirS* in various stress conditions such as the presence of 1/2 MIC of oxacillin for MSSA and 5 μg/ml for MRSA or 1/4 MIC of fusidic acid for MSSA and MRSA [49] on total RNA purified after 30 min of exposition. MICs were determined using CLSI recommendations [50], and the stress experiments were performed with drug concentrations showing minor impact on growth rate following control experiments (S3 Fig).

### Construction of the *tirS*-deleted mutant

*S*. *aureus* RN4220 (lab strain collection) was used for plasmid amplification and genetic manipulations because it is a nitroso-guanidine-induced mutant capable of accepting *E*. *coli* DNA [51]. To delete the *tirS* gene, we performed allelic replacement using double crossover recombination as previously described [52]. Using the primers and restriction enzymes listed in S1 Table, we generated two fragments of 930 bp 5′ and 1029 bp 3′ of *tirS*. These fragments were ligated in 5′ and 3′ of the chloramphenicol acetyl transferase gene [53] and inserted into pMAD [54]. Plasmid and inserts were checked by PCR and sequencing using the primers listed in S1 Table. Plasmid was introduced by electroporation in RN4220 and then in HT20030749 (Geraldine strain). We performed double crossover recombination yielding to deletion of *tirS* in HT20030749 (Geraldine strain). The mutant strain obtained was called Δ*tirS*. Gene deletion was checked by PCR and sequencing using specific primer hybridizing with internal and external positions of the deleted region (S1 Table). Insert presence was checked with primers IngDNA_F and vG_CDS_1_R or IngDNA _F and vG_cat_1_R, yielding a negative PCR and a 2017-bp amplicon for the correct construct.

### Construction of the *tirS* restored strain

Total DNA and plasmid DNA were prepared with Qiagen kits (QIAamp DNA Mini Kit and QIAprep Spin Miniprep Kit) after lysostaphin lyses for *S*. *aureus*. When necessary, transformation of *E*. *coli* DH5α (Promega, Madison, USA) was performed by treatment with CaCl_2_, and *S*. *aureus* strains were transformed by electroporation (Bio-Rad gene pulser). The *tirS*-deleted strain was complemented by inserting the *tirS* gene sequence downstream from the leukocidin promoter P-*lukS* in the bacterial chromosome using sequences homologous to sequence NWMN_0029 and NWMN_0030 of Newman strain (GenBank: AP009351.1) for chromosomal recombination. The region of NWMN_0029 and NWMN_0030 was amplified using primers New29-523 and New30-2371, restricted by EcoRI and SalI and cloned on a pBluescript vector (Stratagene) to obtain plasmid pLUG37. The *tirS* gene sequence was amplified and cloned between the P-*lukS* promoter region and *lukF*-PV transcriptional terminator of the Panton-Valentine leukocidin genes (respectively amplified using primers phi259/phi748 and phi2648/phi2819) on pBluescript. The whole DNA fragment obtained by SmaI was cloned in pLUG37 in the natural EcoRV restriction site between the NWMN39 and NWMN30 sequences. From the resulting plasmid, the DNA fragment corresponding to NMWN30-PlukS-tirS-term lukF-NWMN29 obtained by EcoRI-SalI restriction was cloned in the pMAD vector (pLUG1158). The resulting chromosomic restored strain was called Δ*tirS* + *tirS*. Expression of *tirS* in the restored strain was confirmed by RT-PCR. Oligonucleotides primers for PCR and DNA cloned subfragments are detailed in S2 Table.

### Luciferase activity assay

HEK293T cells (American Type Culture Collection (ATCC), USA) were transiently transfected using Fugene (Roche) for 24 h, according to the manufacturer’s instructions, for a total of 0.4 μg of DNA consisting of 50 ng TLR plasmids, 200 ng of pBIIXLuc, a reporter plasmid containing luciferase under the control of two Igκ-κB sites [55], 5 ng of control Renilla luciferase (pRL-null, Promega), and 50 ng of myc-TirS expression vector, unless stated otherwise. In the case of TLR4, MD2 was co-transfected for efficient detection of LPS. When indicated, an increasing amount of vector (ng) was used for the transfections to obtained different levels of expression of TirS. In all cases, the total amount of DNA was kept constant by adding empty vector. Negative control corresponds to empty vector alone (pCDNA3.1). Where indicated, cells were treated with *E*. *coli* LPS (1 μg/ml), Pam_2_CSK4 (100 ng/ml), CpG ODN1826 (1 μM), and Flagellin Fl-ST (1 μg/ml), all obtained from Invivogen, for 6 h, and then cells were lysed and luciferase activity measured using the Dual-Glo Luciferase Assay System (Promega). In the case of IL-1R and TNFR, endogenous detection was monitored following 6 h of stimulation with IL-1β (100 ng/ml) or TNF-α (100 ng/ml). The *tirS* construct was obtained with the following primers *tirS*-fw-GGGGACAAGTTTGTACAAAAAAGCAGGCTTCTCAGTATTAGAAACTAAATTAAAAAG and *tirS*-rev-GGGGACCACTTTGTACAAGAAAGCTGGGTCCTAATTCTTAGAATTAACGATTACTTG and then cloned in the gateway (Life Technologies) entry vector and subsequently in the pDEST-Myc (Life Technologies) to create an N-terminal myc tag fusion with TirS.

### Immunofluorescence microscopy

HeLa cells (ATCC, USA) were transfected with *myc-tirS* using Fugene (following manufacturer’s instructions) for 10 h. Cells were either fixed in 3% paraformaldehyde, pH 7.4, at room temperature for 15 min or placed in ice-cold methanol for 5 min. Cells were then permeabilized for 10 min with 0.1% saponin in PBS, followed by blocking for 1 h with 2% bovine serum albumin and 10% horse serum in PBS with 0.1% saponin. Primary antibodies were incubated for 1 h followed by three washes in PBS, 1 h incubation for secondary antibodies, two washes in PBS, and one wash in water before being mounted with Prolong Gold. Primary antibodies used were rabbit anti-myc (Abcam) at 1/1000, with either mouse anti-beta tubulin (TUB 2.1) at 1/250 or mouse anti-vimentin (V9) at 1/100 (both Sigma). Secondary antibodies used: donkey anti-rabbit Alexa 488, donkey anti-mouse Alexa 555 (Life Technologies) at 1/1000. The actin cytoskeleton was labeled with phalloidin 568 (Life Technologies). Samples were examined on a Zeiss 710 laser scanning confocal microscope for image acquisition. Images of 1024 × 1024 pixels were then assembled using ImageJ and Adobe Photoshop 7.0.

### Growth curves of *S*. *aureus* strains

*S*. *aureus* Geraldine WT and Δ*tirS* strains were grown in fresh BHI medium or TSB medium at 35°C, after dilution of overnight cultures to OD_600_ = 0.03. A thermostated microplate reader (TECAN M200 Infinite Pro) was used to follow bacterial growth by measuring OD_600_ every 15 min for 24 h. As controls, specific wells were inoculated with medium only. Experiments were done in triplicate.

### Murine model of *S*. *aureus* subcutaneous infection

#### Bacterial isolates and growth

*S*. *aureus* Geraldine WT, Δ*tirS*, and Δ*tirS* + *tirS* strains were used in a murine model of skin infection. For preparation of the inoculum used for subcutaneous inoculation, the bacteria were grown into BHI medium at 37°C for 8 h with constant shaking (200 rpm). They were washed twice and resuspended in sterile PBS, aliquoted to a final concentration of 1.10^7^ bacteria/ml, and stored at -80°C until used. For determination of bacterial titers, samples were serially diluted, plated on agar, and incubated overnight at 37°C. Viability of the inocula was confirmed by colony counts with each experiment. Moreover, to ensure that the results of experiments were consistent, hemolysis phenotype was checked using blood agar plates (Trypcase soy agar + 5% sheep blood, bioMérieux, France).

#### Mouse strains

Mice on a C57BL/6 genetic background were used in all experiments. Six-week-old WT female mice were purchased from Charles River, France. Male and female MyD88-deficient mice were kindly provided by L. Genestier (from S. Akira’s lab; [56]). All mice were maintained in pathogen-free conditions in a biosafety level 2 facility at the Plateau de Biologie Expérimentale de la Souris (PBES, Ecole Normale Supérieure de Lyon, Lyon, France).

#### Skin lesion model

One day prior to infection, mice were prepared for inoculation. Animals first were anesthetized with 2% isoflurane, and a flank was shaved with electric clipper and hair remover cream (Nair, Church & Dwight Co. Inc., Princeton, NJ) on the shaved flank. On the day of infection, mice were infected subcutaneously with 100 μl of the bacterial suspension (1.10^6^ CFUs) in the shaved area. This inoculum was determined in preliminary studies to produce consistent skin lesions. Mice were returned to their cages and observed to awaken. All mice had free access to food and water throughout the duration of the experiments. Animals were weighed at 24 h intervals for 14 days. The area of lesions was measured daily using an electronic caliper and calculated with the formula *A* = *π* × (*L*/2) × (*W*/2) (mm^2^). PBS injection was used for controls.

#### Bacterial recovery and cytokine quantification in skin lesions

To determine the number of CFUs at the site of infection, a second set of mice was inoculated as described above. On days 5 to 9 after infection, mice were euthanized by cervical dislocation; the lesion and the surrounding tissues were removed and transferred in sterile tubes. Tissue samples were weighed, homogenized in 1 ml of PBS (gentleMACS Dissociator, Miltenyi Biotec, Germany), diluted in sterile PBS, and plated on selective agar (ChromID *S*. *aureus*, bioMérieux, France). Enumeration of CFUs was performed 24 h later. For determination of cytokine and MPO levels, lesion homogenates were centrifuged and the supernatant removed and immediately stored at -80°C. Cytokine levels were determined using Luminex assays (Bio-Techne) according to the manufacturer’s instructions. The amount of MPO in the skin lesions was quantified using a commercially available ELISA kit (Bio-Techne).

### Statistical analysis

The data were analyzed using R software (http://www.r-project.org). Student’s t test, Wilcoxon test, or one-way ANOVA followed by multiple comparisons tests (Tukey) were used to compare *tirS* expression, luciferase activity, lesion size, cytokine levels, and bacterial CFUs between the groups. Correlations were assessed by Spearman’s correlation. In all experiments, values of * p < 0.05, ** p < 0.01, and *** p < 0.001 were considered statistically significant.

## Supporting Information

S1 Table Primers and restriction enzymes used for construction of the *tirS*-deleted strain. (PDF)

S2 Table Primers and restriction enzymes used for construction of the *tirS*-restored strain. (PDF)

S1 Fig Intracellular localization of ectopically expressed GFP-TirS in HeLa cells. (TIF)

S2 Fig Growth curves of *S*. *aureus* Geraldine WT and Δ*tirS* in BHI medium or TSB medium. (TIF)

S3 Fig Growth curves of *S*. *aureus* strains in presence of oxacillin or fusidic acid. (TIF)
