# Correction: The TIR Homologue Lies near Resistance Genes in *Staphylococcus aureus*, Coupling Modulation of Virulence and Antimicrobial Susceptibility

**DOI:** 10.1371/journal.ppat.1006291

**Published:** 2017-03-24

**Authors:** Sabine Patot, Paul R. C. Imbert, Jessica Baude, Patricia Martins Simões, Jean-Baptiste Campergue, Arthur Louche, Reindert Nijland, Michèle Bès, Anne Tristan, Frédéric Laurent, Adrien Fischer, Jacques Schrenzel, François Vandenesch, Suzana P. Salcedo, Patrice François, Gérard Lina

There is an error in the XML that is causing the 2nd and 14th authors’ names, Paul RC Imbert and Suzana P Salcedo to be indexed incorrectly and affects the citation. The names should be indexed as Imbert PR and Salcedo SP. The correct citation is: Patot S, Imbert PR, Baude J, Martins Simões P, Campergue J-B, Louche A, et al. (2017) The TIR Homologue Lies near Resistance Genes in *Staphylococcus aureus*, Coupling Modulation of Virulence and Antimicrobial Susceptibility. PLoS Pathog 13(1): e1006092. doi:10.1371/journal.ppat.1006092
