# Supplementary material for: The TIR Homologue Lies near Resistance Genes in Staphylococcus aureus, Coupling Modulation of Virulence and Antimicrobial Susceptibility
Source: PLoS Pathog. 2017 Jan 6;13(1):e1006092. doi: 10.1371/journal.ppat.1006092 (PMC5218399; doi:10.1371/journal.ppat.1006092)
Supplement: S1 Table — (PDF) [file ppat.1006092.s001.pdf]

S1 Table. Primers and restriction enzymes used for construction of the *tirS*-deleted strain.

Primer	5′–3′ sequence	Restriction enzyme
tirS_6345-6364_F	ATAGGGATCCACTAAAATTCCGAAACCACA	BamHI
tirS_7246-7275_R	TCTACCGCGGATAATACCTCGCTTTTTATA	SacII
tirS_8119-8151_F	ACGACCTAGGTAATAGTATTGGTTATCTACTTT	AvrII
tirS_9119-9148_R	AAATGGAATTCAACAGTTACTCCCGCTTCT	EcoRI
vG_CDS_1_R	AATCTCTTCATTTCCTTTTGTATTTTTATCT	
vG_CDS_2_F	GATTATGGAAAAATTATTAACTAAT	
vG_cat_1_R	GAAAGCAGACAAGTAAGCCTCCTAAA	
vG_cat_2_F	GAAAGAGAAAAAGCATTTTCAGGTA	
vG_H1_F	AACTTATAGTGATTGGGATATGCTA	
vG_H2_F	AGAATTAAATTCAATTAATGAATA	
vG_H3_F	GGAATTAAATGAAAAAAGCAGTGT	
vG_H4_F	ACTAATCTATTGAACGAACCCCATTA	
vG_H5_R	ACTATTTCATACTCATCAATCGAT	
vG_H6_R	TAATTCCCTCTTTGCTTTTAAAGTAG	
vG_H7_R	TAATACCTCGCTTTTTATAATCTT	
vG_H8_R	CTATAAGTTTCTTTTAAAAATATC	
IngDNA_F:	TACCTTGATCAATAAACTCATTTAATAAGTCTT	
IngDNA_R:	ATCTTCCATACTATCTTAGCAGATGATTTTAT	
