# Supplementary material for: The TIR Homologue Lies near Resistance Genes in Staphylococcus aureus, Coupling Modulation of Virulence and Antimicrobial Susceptibility
Source: PLoS Pathog. 2017 Jan 6;13(1):e1006092. doi: 10.1371/journal.ppat.1006092 (PMC5218399; doi:10.1371/journal.ppat.1006092)
Supplement: S2 Table — (PDF) [file ppat.1006092.s002.pdf]

Primer	5′–3′ sequence	Restriction
phi259	TTACCCGGGTAATAGTGGACAACGTCG	SmaI
phi748	CATGATATCATTTCCTTTCTTTATAAATTTTATTAC	EcoRV
phi2648	AAATCCTGGATCCTAAACAGATAGATAATCAAAAAA	BamHI
phi2819	TTCCCGGGTTCCACAAGTGTTTGCC	SmaI
tir391	AGGAGGCCTATGTCAGTATTAGAAAC	StuI
tir1250	TACGGATCCTAATTCTTAGAATTAAC	BamHI
New29-523	GAGAATTCAGTGGCTACATTCGAACATATC	EcoRI
New30-2371	GCGTCGACTTAAATATGGGATGTCC	SalI
tirS-F RT-PCR	AGTGGTTGGAGTAGGTACGA	
tirS-R RT-PCR	GCACGTACATCTTCAACACTCA	
