# Supplementary figures and images for: The TIR Homologue Lies near Resistance Genes in Staphylococcus aureus, Coupling Modulation of Virulence and Antimicrobial Susceptibility
Source: PLoS Pathog. 2017 Jan 6;13(1):e1006092. doi: 10.1371/journal.ppat.1006092 (PMC5218399; doi:10.1371/journal.ppat.1006092)

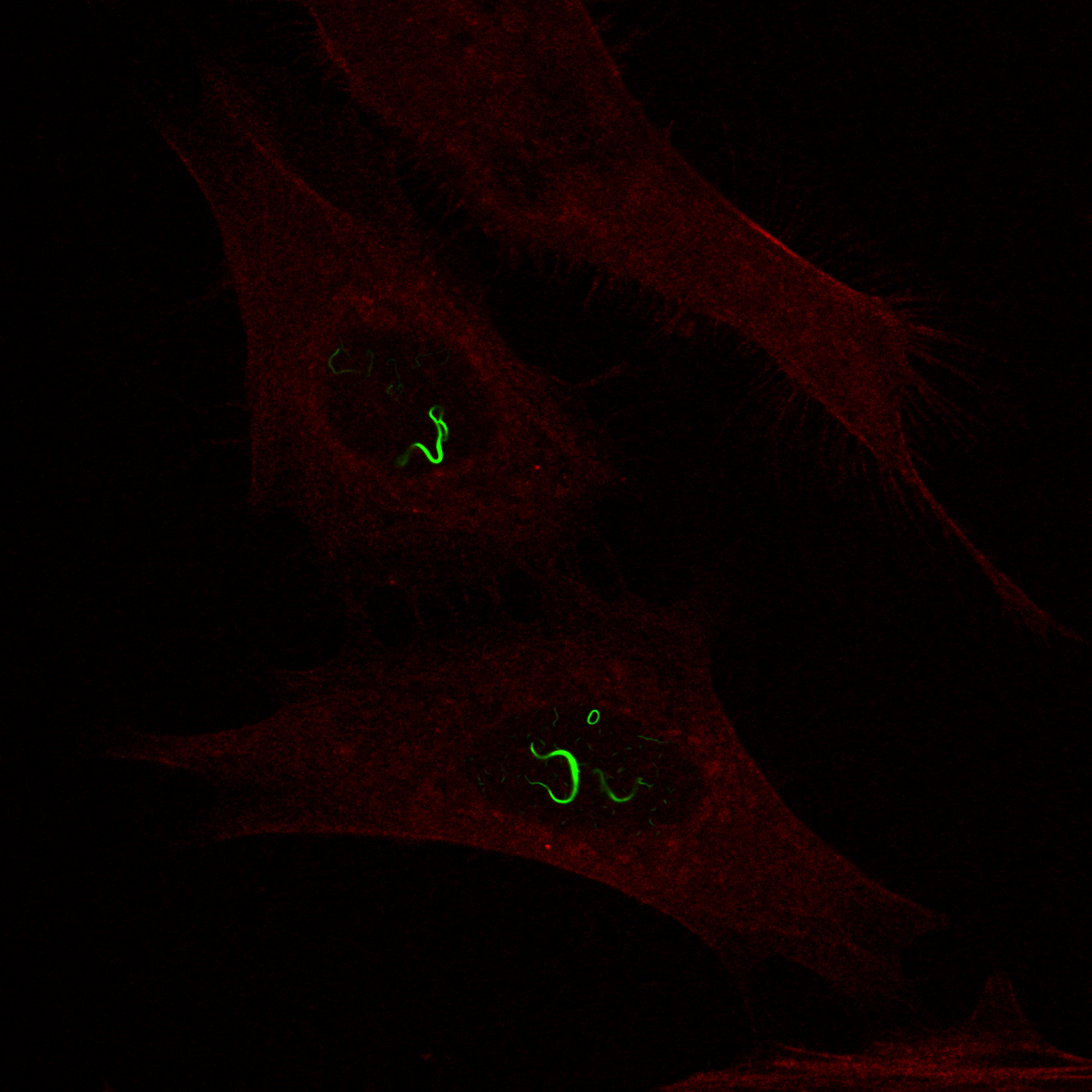

Supplement: S1 Fig — (TIF) [file ppat.1006092.s003.tif]

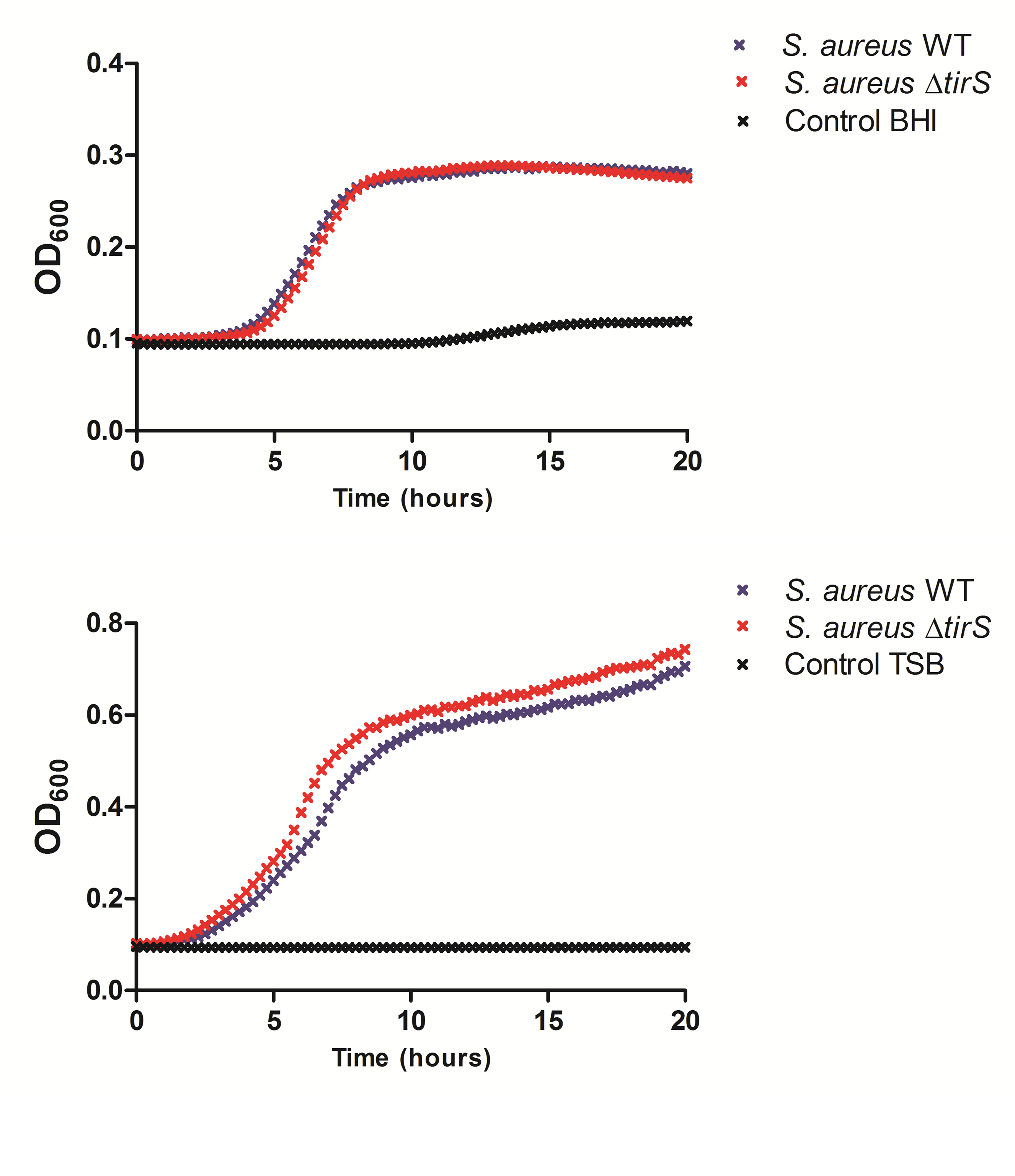

Supplement: S2 Fig — (TIF) [file ppat.1006092.s004.tif]

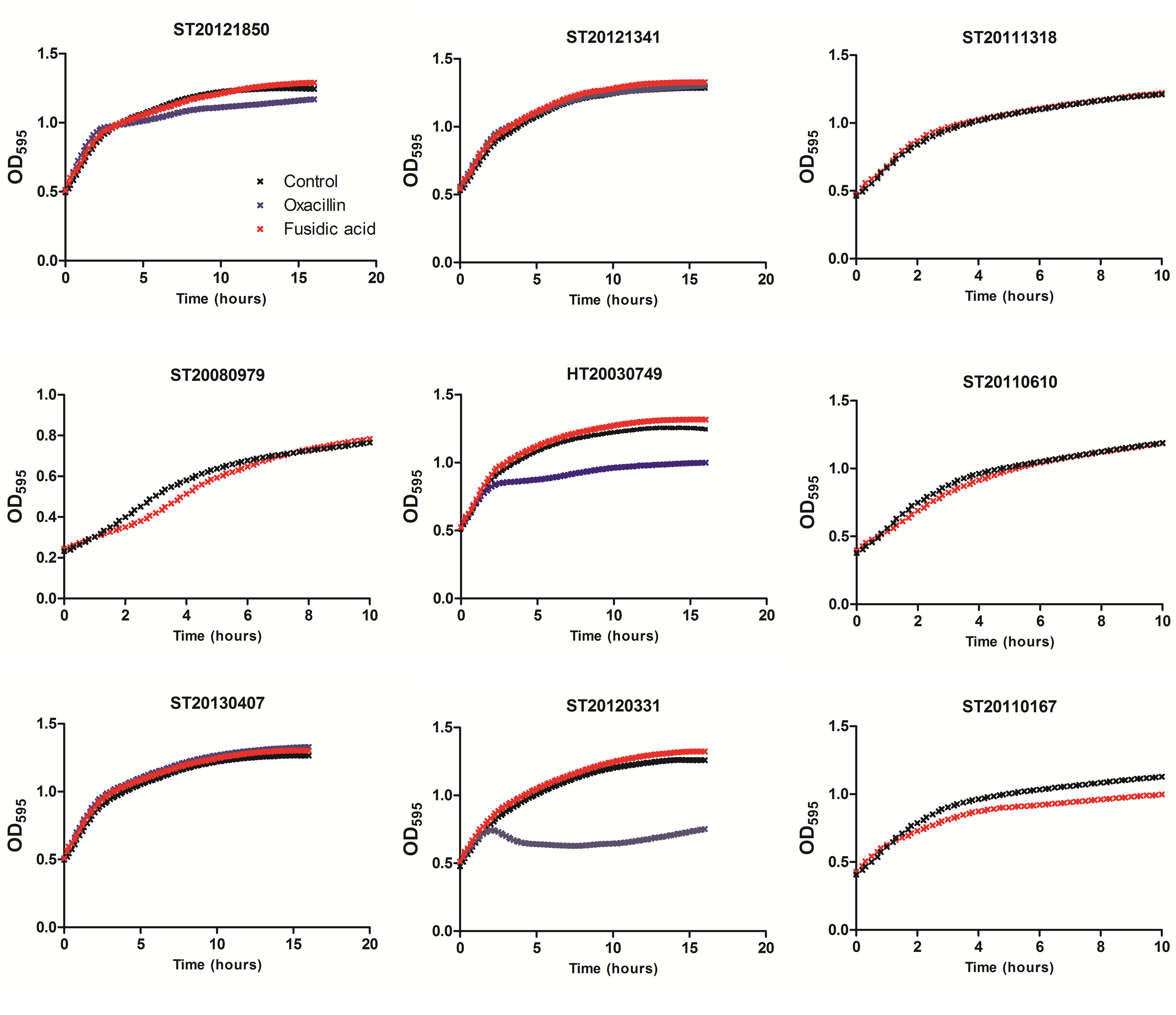

Supplement: S3 Fig — (TIF) [file ppat.1006092.s005.tif]
